# Avoiding unconscious injection of vial-derived rubber particles during intra-articular drug administration

**DOI:** 10.1016/j.ocarto.2021.100164

**Published:** 2021-04-04

**Authors:** Andreas Hecker, Agostino Di Maro, Emanuel F. Liechti, Frank M. Klenke

**Affiliations:** Department of Orthopaedic Surgery and Traumatology, Inselspital, Bern University Hospital, University of Bern, Freiburgstrasse 4, 3010, Bern, Switzerland

**Keywords:** Vial coring, Intra-articular injections, Filter needle, Drug contamination, Foreign body reaction

## Abstract

**Objective:**

Vial coring describes the occurrence of small rubber particles, which are formed by needles when perforating vial stoppers. These particles may be aspirated along with the drug. Unconscious injection of rubber particles may increase the risks associated with intra-articular injections. This study aimed to analyze the frequency of this phenomenon and possibilities to avoid its occurrence.

**Method:**

800 vials of 2 ​mL, filled with sodium chloride, were divided into 4 groups (n ​= ​200 each). Aspiration through the rubber stopper was performed with a 18-Gauge needle and the fluid was ejected onto a 10 ​μm filter paper through a 18-Gauge needle (group one) and a 23-Gauge needle (group two). In group three a 23-Gauge needle was used for aspiration and ejection. In group four, aspiration was performed using 18-Gauge needles with implemented 5 ​μm filters. Subsequently, a microscopic analysis of the filter papers was performed.

**Results:**

In none of the 800 specimen, a rubber particle was detected by naked eye. Microscopically, 20 (10%) rubber particles were detected in group one, 21 (11%) in group two and 65 (33%) in group three. In group four, no particles were visualized.

**Conclusion:**

This study shows the occurrence of rubber particles in 10–33% of the cases, when standard needles are used for the aspiration of drugs. We therefore recommend using industrially prefilled syringes, filter needles or removing the rubber stopper before withdrawing drugs from vials for intra-articular injections.

## 1 Introduction

Intra-articular injections for therapeutic and diagnostic reasons with local anesthetics and corticosteroids represent an established clinical practice, commonly used in the symptomatic treatment of inflammatory rheumatic diseases and osteoarthritis [[1], [2], [3], [4], [5], [6], [7], [8]].

Complications of intra-articular injections include local hematoma, infection, hypopigmentation, subcutaneous fat and muscle atrophy and osteonecrosis [9,10], the most devastating being septic arthritis. A complication that has not been reported frequently and the clinical effects of which are not clear, is the inadvertent injection of small rubber particles into the joint. The occurrence of such a foreign body in the injection fluid is called vial coring and is reported with an incidence between 4 and 40% in in-vitro studies depending on the applied needle and puncture angle [[11], [12], [13], [14]].

Vial coring describes the occurrence of small rubber particles, which are formed by needles when perforating vial stoppers. These particles may be aspirated from the vial into the syringe along with the drug, and could therefore be injected into joints unconsciously [15]. There are no published reports on intra-articular injection of rubber particles available to date. However, embolisms after intravenous drug application was found to be related to vial coring, indicating that intra-articular injections are at risk for vial coring associated complications including septic arthritis [11,[16], [17], [18], [19]]. Moreover a foreign body reaction could promote rapid destructive osteoarthritis [20].

An effective way to reduce vial coring by about 50% is an insertion angle of the aspiration needle into the vial at 45–60° [21]. Unfortunately, this is difficult to implement into routine orthopedic practice due to safety reasons. Furthermore, a decrease of vial coring by 50% may not be sufficient.

We designed this study to evaluate methods to avoid vial coring. Our hypothesis was that the injection of rubber cores into joints can be reduced significantly by injecting the drug through a thinner needle than the one used to withdraw the liquid from the vial. We also hypothesized that the use of needles with a filter for aspiration from the vial may avoid vial coring completely.

## 2 Method

Vials with a volume of 2 ​mL (DWK Life Science, Wertheim, Germany; 10472752) and the corresponding standard rubber stoppers were used for this experiment (total n ​= ​800, n ​= ​200 per group). Each vial was filled with 1 ​ml sterile sodium-chloride solution under sterile conditions and closed with a rubber stopper. Aspiration was performed through the stopper with the needle inserted exactly in the center at an angle of 90°.

In group one, aspiration from the vials was performed with a standard 18-Gauge (G) needle (Sterican®, B. Braun, Melsungen AG, 34209 Melsungen, Germany), and the same needle was used to eject the aspirated fluid onto a 10 ​μm filter paper (Fisher Scientific AG, Reinach, Switzerland; 11718553) of 2 ​× ​2cm. In group two, an 18-G needle was used for aspiration. The needle was then replaced for a 23-G needle (Sterican®, B. Braun, Melsungen AG, 34209 Melsungen, Germany) through which the fluid was ejected onto the filter paper. In group three a 23-Gauge needle was used for aspiration and ejection. In group four, aspiration was performed using 18-G needles with implemented 5 ​μm filters (Blunt Fill Needle with Filter, BD, Franklin Lakes, USA). Afterwards the needle was removed and the fluid ejected onto the filter paper directly out of the syringe.

The filter papers were transferred onto a transparent microscope slide. For quantification of rubber particles adhesive microscope slide grids (Merck KGaA, Darmstadt, Germany, Z688533, 2 ​× ​2 cm, grid distance 1 ​mm) were used.

Microscopic investigation and quantification of rubber particles was performed under a light microscope (Eclipse E800, Nikon AG, 8132 Egg, Switzerland) equipped with a digital camera system (DS-Fi3, Nikon Corporation, Japan). The entire area of the slide grips was evaluated for rubber particles. First, each 1 ​× ​1 mm square was examined under 10-fold magnification and scanned for particles. If a particle was detected, 20-fold magnification was used to measure its length and width. The total number of particles per group was noted.

Simple descriptive statistics (percentages, mean, minimum and maximum) was performed.

## 3 Results

In none of the 800 specimen, a rubber particle was detected by naked eye. Microscopically, 20 (10%) rubber particles were detected in group one, in which the 18-G needle was used for aspiration and ejection. In group two, in which 18- and 23-G needles were utilized, 21 (10.5%) particles were found. In group three, in which a 23-G needle was used for aspiration as well as for ejection 65 (33%) rubber particles were seen. In group four, where the 18-G needle with 5 ​μm filter was used for withdrawal of the fluid from the vial no particles were visualized.

**Fig. 1:**
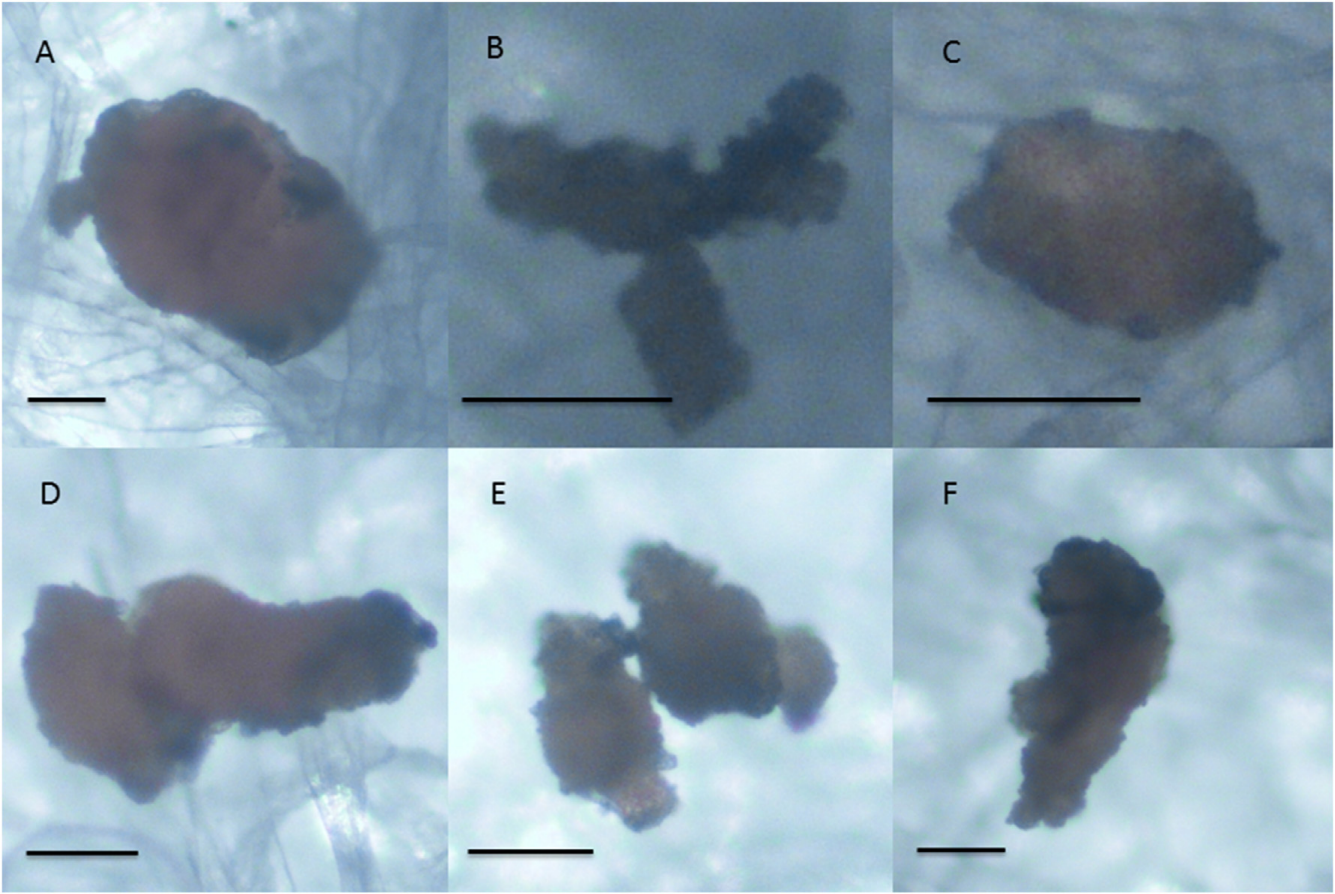
Microscopically detected rubber particles. Examples of the detected cores from group one (A–C) and group two (D–F). 2–4 particles tended to group on the filter paper (B, D, E). Scale 50 ​μm.

The detected particles were mostly cylindrical with a mean length of 77 ​μm ranging from 29 to 214 ​μm. The mean width was 36 ​μm with a range from 14 to 83 ​μm in groups one and two were the 18-G needle was used for aspiration. In group three, in which the 23-G needle was used for aspiration the particles had a mean length of 55 ​μm ranging from 21 to 121 ​μm and a mean width of 32 ​μm with a range from 13 to 72 ​μm. Fig. 1 shows examples of the detected cores.

## 4 Discussion

In this experiment, we found a coring rate of 10% when a 18-G needle without a filter was used for aspiration, independent of the needle diameter used for fluid ejection. Using a 23-G needle for aspiration led to a coring rate of 33%. Rubber particles in the injection fluid could be completely avoided by using a needle with 5 ​μm filter to withdraw the fluid from the vial.

Therefore, our first hypothesis, that coring may be reduced if a thinner needle is used for injection than for aspiration could not be confirmed. Using a needle with a 5 ​μm filter for fluid withdrawal eliminated vial coring completely, confirming the second hypothesis of this study.

The data of this study shows that the term “coring” is somewhat misleading. One would expect a particle of the size of the inner diameter of the needle. In fact, the inner diameter of the 18-G needle used in this study was 950 ​μm and that of the 23-G needle was 390 ​μm. The rubber particles created by the 18-G needle had a mean diameter of 77 ​μm with a maximum of 214 ​μm. Therefore, even the largest particles could easily pass the smaller diameter injection needle used for group two. The small size of the rubber particles is most likely caused by stretching of the rubber stopper when the needle is inserted and subsequent contraction of the resulting core.

Interestingly, using a thinner 23-G needle for aspiration resulted in a threefold coring rate. This result is in accordance with the available literature, as Asakura et al. found a coring rate of 73% in primary aspirations of insulin through a rubber stopper using a 31-G needle [22]. This data and our results suggest that thinner needles lead to higher coring rates.

The clinical relevance of injecting small rubber particles as foreign bodies into joints has not been investigated. However, it seems reasonable to assume that vial coring is a risk for septic arthritis subsequent to intra-articular injections. Furthermore, it may be linked to rapid destructive osteoarthritis (RDO) via a foreign body reaction [23]. The exact cause of this condition remains to be elucidated but the disease has been associated with intra-articular injections of the hip and knee joint [24]. In this regard, a single center study of 111 patients with RDO of the knee found that 88% had an intra-articular injection before developing this condition [25].

Many studies investigated the reaction of human tissue especially synovial tissue to different biodegradable and not biodegradable substances. A foreign body reaction is often found after usage of both material groups and leads to a high number of multinucleated foreign body giant cells, macrophages as well as to the expression of collagenases and proteases [20,26]. Synovial tissue seems to be prone to generate this reaction. Also a generalized synovitis with lymphoplasmatic infiltration was reported [[27], [28], [29]]. Especially the macrophages release pro-inflammatory mediators like prostaglandins and many others after phagocytosis of foreign bodies [30]. This inflammation can finally lead to chondrolysis, bone resorption and end stage osteoarthritis [31,32]. Proteases released during this inflammatory process are mainly responsible for damaging the cartilage [33].

Patients with RDO were found to have higher levels of inflammatory cells especially proteolytic enzymes compared to patients with “normal” coxarthritis. This cells are the same as found in foreign body reactions [34]. The causes of the RDO remain unclear, thus many theories exist. Mechanical, immunological and toxic reasons haven been discussed. The final result is a fulminant inflammation with cartilage and bone destruction [23]. Next to the proposed reasons the latter could also be induced by a foreign body reaction as outlined above. The massive bone loss that is often seen in RDO is result of a high count of osteoclast, which differentiate from macrophages [35]. Given that macrophages are important in the genesis of osteoarthritis and the fact that they are found in very high counts in foreign body reactions, the latter has to be considered a realistic risk factor for developing RDO [36].

To reduce vial coring a needle insertion angle of 45–60° [21] has been proposed. However, this is not applicable in orthopedic daily practice. A nurse usually holds the vial while the surgeon aspirates fluid with sterile gloves. There is an increased risk of slipping and stitch injury if the needle is not administered in a 90° angle. Furthermore, merely reducing the number of particles does not seem to be sufficient with the existence of filter needles. Such needles provide a practicable and inexpensive alternative to completely avoid vial coring.

Unfortunately many corticosteroids used in daily orthopedic practice are particulate with a particle size of up to 100 ​μm [37]. This makes the use of filter needles impossible for this kind of drugs. Glass vials without rubber stoppers are also seen controversial because glass delamination particles have been described and therefore filter needles are also recommended [38,39]. To avoid vial coring, filter needles can be used along with non-particulate corticosteroids. Another acceptable solution is the removal of the rubber stopper and directly withdrawing the drug out of the vial if particulate drugs are used. For this task, decapping tongs are available for the common vial sizes. Moreover industrially manufactured syringes already filled with the respective drugs exist. This study outlines that foreign bodies are frequently injected into joints, when drugs are withdrawn through rubber stoppers. Therefore, we recommend to avoid this by applying one of the above suggested solutions.

This study has limitations because sodium chloride was used instead of cortisone or local anesthetics due to costs. However, a different coring rate is not expected with a different medium. The 10 ​μm filter paper only allows detection of particles larger than this size, so there could be smaller particles present that could increase the coring rate. The major limitation is that adverse effects of rubber particles cannot be proven by this study. Nonetheless, the authors note in this section by reviewing the literature, that there is a potential risk from foreign body reactions and therefore recommend avoiding vial coring whenever possible.

This study shows the occurrence of rubber particles in the injection fluid in 10–33% of the cases, when standard needles are used for the aspiration of drugs. We therefore recommend using industrially prefilled syringes, filter needles or removing the rubber stopper before withdrawing drugs from vials for intra-articular injections.

## Author contributions

AH and ADM performed the measurements and prepared the manuscript draft. EFL analyzed the data and corrected the manuscript. FMK supervised and finalized the manuscript. All authors reviewed the final manuscript and agree to be responsible for all aspects of the work.

## Funding

Not applicable.

## Declaration of competing interest

The authors, their immediate family, and any research foundation with which they are affiliated did not receive any financial payments or other benefits from any commercial entity related to the subject of this article. None of the authors has a conflict of interests.
